# Sleep cycle in children with severe acute bronchopneumonia during mechanical ventilation at different depths of sedation

**DOI:** 10.1186/s12887-022-03658-8

**Published:** 2022-10-12

**Authors:** XueShan Zhao, JingLi Yan, Bo Wu, Duo Zheng, Xiuying Fang, Wei Xu

**Affiliations:** 1 Department of Pediatrics, Shengjing Hospital of China Medical University, No.36, San Hao Street, Heping District, Shenyang, LiaoNing Province China; 2 Department of Nerve Function, Shengjing Hospital of China Medical University, Shenyang, China

**Keywords:** Sleep cycle, Sedation, Pediatric patients, Pneumonia, Mechanical ventilation, Intensive care

## Abstract

**Background:**

To investigate the characteristics of sleep cycle in children with severe acute bronchopneumonia treated with invasive mechanical ventilation at different sedation depths.

**Methods:**

We included 35 pediatric patients with severe acute bronchopneumonia treated using mechanical ventilation in Pediatric Intensive Care Unit of Shengjing Hospital of China Medical University. They were divided into deep sedation group (*n* = 21; ramsay score 5–6) and light sedation group (*n* = 14; ramsay score3-4) based on sedation depth achieved during mechanical ventilation. Long-term video electroencephalography (EEG) monitoring was performed within the first 24 h after starting mechanical ventilation and after weaning from mechanical ventilation and discontinuing sedatives and analgesics. The results were analyzed and compared with those of normal children to analyze changes in sleep cycle characteristics at different sedation depths and mechanical ventilation stages.

**Results:**

There were 29 cases altered sleep architecture. The deep sedation group had a significantly higher incidence of sleep architecture altered, total sleep duration, and non-rapid eye movement sleep-1 (NREM-1) loss incidence than the light sedation group. Moreover, the deep sedation group had a significantly lower awakening number and rapid eye movement sleep (REM) percentage than the light sedation group. The sleep cycle returned to normal in 27 (77%) patients without NREM-1 or REM sleep loss.

**Conclusions:**

Deep sedation during mechanical ventilation allows longer total sleep duration, fewer awakenings, and an increased deep sleep proportion, but sleep architecture is severely altered. After weaning from mechanical ventilation and sedative discontinuation, lightly sedated children exhibit better sleep recovery.

**Supplementary Information:**

The online version contains supplementary material available at 10.1186/s12887-022-03658-8.

## Background

Sleep disruption are common among patients in the intensive care unit (ICU); moreover, they are easily overlooked as treatment complications in critically ill patients. Sleep monitoring has revealed different sleep rhythms and sleep cycle levels in most pediatric intensive care unit (PICU) patients [1]. Severe disease condition state is the most important factor affecting sleep in critically ill children. In addition to direct injury to brain sleep centers caused by intracranial lesions, sleep is also affected by physical and psychological pain caused by dysfunction of other important organs, especially diseases affecting brain perfusion, oxygen supply, and metabolic substrate supply, including shock, heart failure, respiratory failure, acute respiratory distress syndrome, hypoglycemia, and other metabolic crises [2–4]. Noise in the PICU, irregular light cycles, invasive operations and treatments, and routine care also affect sleep in critically ill children [5]. Adult studies have been proposed that sleep disruptions and atypical sleep incidence is higher among critically ill patients on mechanical ventilation than in those not on mechanical ventilation [6, 7]. Because routine sleep monitoring is difficult to use in critically ill children, especially in mechanical ventilation states, sleep studies under mechanical ventilation in critically ill children are currently lacking. There is a need to implement sedation and analgesia for children requiring mechanical ventilation to improve comfort, implement better protective strategies for pulmonary ventilation, allow extensive mitigation of unpleasant experiences, improve patient-ventilator synchrony, and prevent unplanned extubation [8–10]. Respiratory failure, invasive mechanical ventilation, and use of sedatives and analgesics may decrease sleep quality in children [11].

Sedation needs differ among children due to differences in the tolerance level for mechanical ventilation [12]. Deep sedation can improve patient-ventilator synchrony and reduce stressful stimuli during various invasive operations. In adult patients with acute respiratory distress syndrome (ARDS) deep sedation could improve lung compliance and oxygenation and reduce ventilator-related lung injury [13]. Under light sedation, the patient’s cough reflex is better maintained and sputum is more easily cleared, which could shorten the mechanical ventilation duration and decrease the incidence of ventilator-related pneumonia and delirium [14]. Few studies exist on the effects of different sedation depths on sleep. There is a need for studies on changes in the physiological sleep cycle under different sedation statuses after weaning from mechanical ventilation and sedative discontinuation and sleep recovery after different sedation depths in children with severe pneumonia on mechanical ventilation. Effective sleep quality monitoring in these children could help clinical personnel provide better sleep environments for critically ill children, reduce human factors for sleep abnormalities. We sought to investigate the characteristics of sleep cycle in children with severe acute bronchopneumonia treated with invasive mechanical ventilation at different sedation depths by long-term video electroencephalography (EEG) monitoring.

## Methods

### Research participants

The study was approved by the Ethics Committee of Sheng Jing Hospital of China Medical University (2020PS607). Informed consent was obtained from patients’ parents before survey. Data were collected from a prospective cohort study on pediatric patients with severe acute bronchopneumonia admitted to the PICU of Sheng Jing Hospital of China Medical University and treated with invasive mechanical ventilation between Jan 2019 and Jan 2020. The inclusion criteria were as follows: (1) diagnosis of severe acute bronchopneumonia requiring invasive mechanical ventilation through endotracheal intubation; (2) age 6 month to 13 years; (3) informed consent from family members; and (4) continuous midazolam sedation and/or sufentanil analgesia. The exclusion criteria were as follows: (1) central nervous system (CNS) infection, cerebral hemorrhage, or head trauma; (2) confirmed brain death or receiving cardiopulmonary resuscitation; (3) admission to the hospital for poisoning or trauma; (4) altered consciousness state before mechanical ventilation and Glasgow score < 11; (5) inability to normally complete examination or unreadable electroencephalography (EEG) results; and (6) death within 24 h of mechanical ventilation initiation.

Finally, we included 35 patients based on the inclusion and exclusion criteria. We recruited 105 children with routine developmental assessments in developmental pediatrics as the healthy control group. Children in healthy control group have no neurological or psychological diseasesor chronic disease status (heart disease, kidney disease, gastrointestinal tract disease, chronic lung disease, etc.), and their growth and development in the same age of children of the same gender belong to the normal range. After communication with parents, we performed 24 h of dynamic EEG without cost. The monitoring time is from 8–9 a. m. to the same time the next day, during which the child's normal life and study are not restricted. The ethics committee agreed and the parents (guardians) were informed and agreed.

### Methods

Monitoring and groups: Vital signs, ECG, and long term video EEG were monitored; further, sedation scores were assigned. The time periods were grouped into two phases as follows: phase 1, the first 24 h after starting mechanical ventilation; phase 2, the first 24 h after weaning from mechanical ventilation and discontinuation sedatives and analgesics. We divided children with ramsay score fluctuation of 3–4 into the light sedation group and children with ramsay score fluctuation of 5–6 into the deep sedation group. The control group comprised ambulatory EEG results of children without sleep patholgy like sleep apnea from January 1, 2015, to December 31, 2018, in our hospital’s outpatient clinic, who were matched by age and gender at a ratio of 1:1.

Mechanical ventilation and sedation: We used a ventilator (Evita 2 Dura, Dräger, Germany) in the BIPAP (biphasic positive airway pressure) mechanical ventilation mode. All the included patients received sufentanil for analgesia at the start of mechanical ventilation. The sufentanil loading and maintenance doses were 0.3–0.5 µg/kg and 0.03–0.05 µg/kg.h-1, respectively. All patients underwent midazolam sedation; moreover, patient-ventilator dyssynchrony was minimized during the monitoring period by adjusting the sedative dose. The midazolam loading and maintenance doses were 0.1–0.3 mg/kg and 2–8 µg/kg.min-1, respectively. The medication was continuously pumped using a micropump. Ramsay scores [15] were obtained within 30 min from EEG monitoring initiation and recorded at subsequent 4-h intervals.

EEG connection and reading of results: the NicoletOne EEG system was used for continuous EEG monitoring. After the head was cleaned, scalp electrodes were applied according to the international 10–20 system. ten bipolar longitudinal channels (C3–C4, O1–O2, F1–F2, P3–P4, T3–T4) were used; moreover, the system was recorded in waveform form through conversion. Two attending physicians from the Department of Functional Neurology interpreted the readings based on the standards of the American Academy of Sleep Medicine (AASM) [16] Sleep Scoring Manual 2.3–2.6. Moreover, they recorded the duration of the rapid eye movement sleep (REM), non-rapid eye movement sleep-1 (NREM-1), NREM-2, and NREM-3 stages and the corresponding percentages (REM%, N1%, N2%, and N3%). Further, they calculated the percentage of total sleep duration during the recording period, and analyzed the intra-sleep characteristics. The sleep durations from 7:00 to 19:00 and from 19:00 to 7:00 on the next day were recorded as the day and night sleep duration, respectively.

Results were considered unreadable if ≥ 2 electrodes fell off or key electrodes fell off that interfered with the image or were significantly affected by interference from other instruments.

### Statistics

Statistical analyses were performed using SPSS 24.0 statistical software. Count data are expressed as the number of cases and rate. The chi-square test was used for between-group comparisons; moreover, the Pearson’s c2 and Fisher’s exact probability tests were used for the total sample size and theoretical number per class, respectively. Normally distributed measurement data are expressed as mean ± standard deviation (SD) after the initial assessment of the variance for homogeneity. The t-test and t’-test were used for data with homogeneous and non-homogeneous variance, respectively. Statistical significance was set at *p* < 0.05.

## Results

### General characteristics

**Fig. 1:**
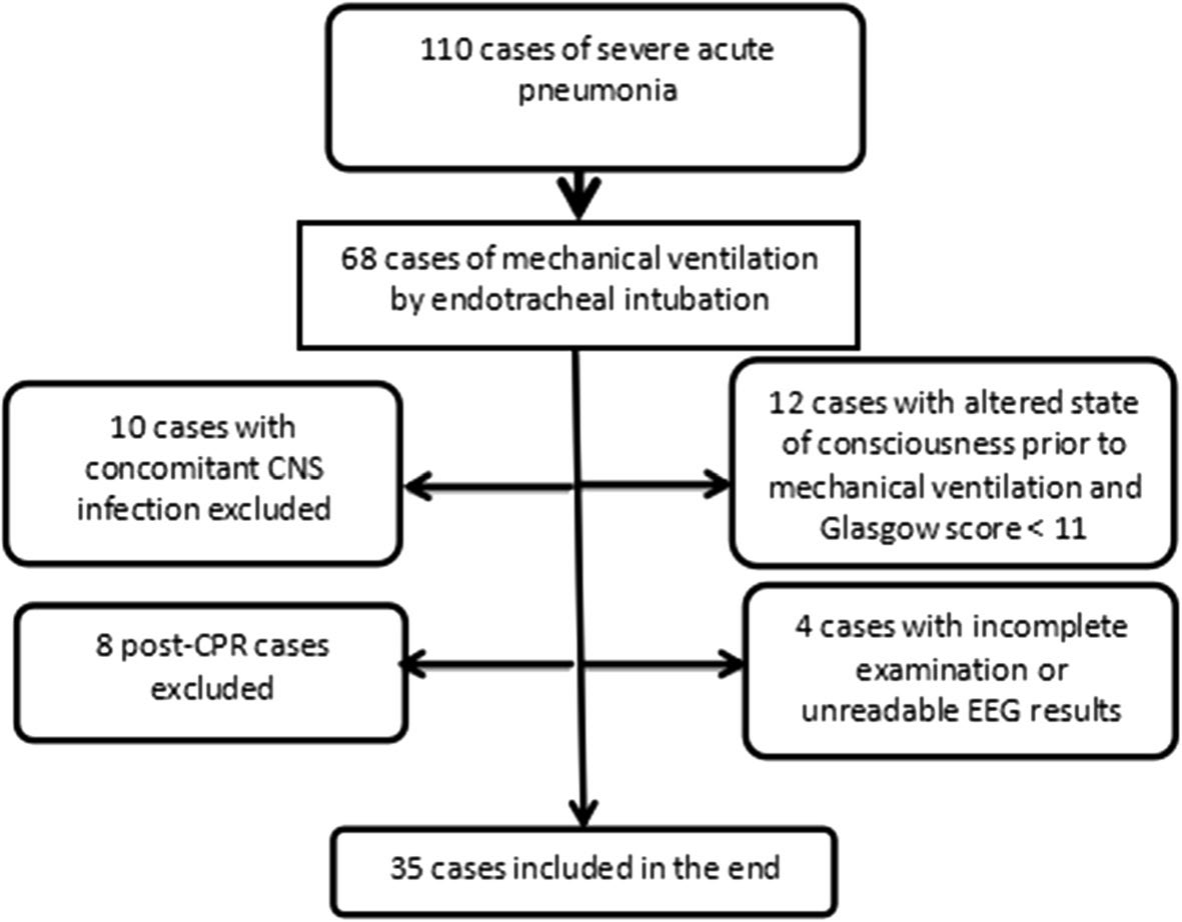
Flowchart of inclusion criteria

There were 35 patients (age range: 6 months to 13 years) with 21 males and 14 females. The cases include process is shown in Fig. 1, There were 2 death cases during mechanical ventilation. None of the patients received analgesics during the monitoring period. Table 1 shows the general characteristics of the patients. Compared with the light sedation group, the deep sedation group showed significantly higher blood partial pressure of carbon dioxide (86.14 ± 28.31 mmHg vs. 66.28 ± 21.86 mmHg; *p* < 0.05) and sedative treatment dose and duration (*p* < 0.05). The deep sedation group had a significantly longer duration of mechanical ventilation than the light sedation group (*p* < 0.05).

**Table 1:** General characteristics of patients in the light and deep sedation groups

Variable	Light sedation group (*n* = 14)	Deep sedation group (*n* = 21)	Test statistic (T/χ2)	*P*-value
Age (years)	3.44 ± 2.90	4.25 ± 3.36	0.914	0.370
M/F (n)	7/7	14/7	0.972	0.324
Hospital stay (days)	14.14 ± 11.73	18.62 ± 10.41	-1.185	0.245
PELOD-2	4 (3–6)	5 (4–6)	0.048	0.275
Type I respiratory failure	2 (14.3%)	3 (14.3%)	0	1
Type II respiratory failure	12 (85.7%)	18 (85.7%)		
ARDS	4 (28.6%)	7 (33.3%)	0.088	0.766
Sepsis	2 (14.3%)	5 (23.8%)	0.476	0.490
MODS	1 (4.8%)	2 (14.3%)	0.972	0.324
Lung imaging			3.889	0.274
Bilateral diffuse inflammation, partial consolidation	6 (42.9%)	12 (57.1%)		
Lobar consolidation and atelectasis	4 (28.6%)	1 (4.8%)		
Bilateral diffuse effusion	2 (14.3%)	4 (19.0%)		
Bronchial stenosis and occlusion	2 (14.3%)	4 (19.0%)		
Concomitant pleural effusion	4 (28.6%)	4 (19.0%)	0.432	0.398
Concomitant pneumothorax, subcutaneous emphysema	1 (7.1%)	4 (19.0%)	0.972	0.321
Duration of mechanical ventilation (h)	123.14 ± 96.52	193.38 ± 162.14	6.359	0.017
Midazolam (ug/kg.d-1)	5588.14 ± 1370.11	8756.14 ± 5022.65	-2.380	0.023
Duration of sedation (d)	5.12 ± 4.03	8.03 ± 6.77	-1.592	0.017
WBC (*10^9^/L)	13.5 ± 10.15	13.8 ± 11.05	-0.087	0.937
C-reactive protein (mg/L)	65.18 ± 66.49	49.45 ± 49.02	0.806	0.426
Procalcitonin (ng/mL)	6.14 ± 4.33	29.54 ± 23.49	-1.376	0.182
Blood lactate (mmol/L)	2.56 ± 1.18	2.78 ± 1.87	-0.385	0.703
PaO_2_ (mmHg)	66.22 ± 16.81	66.14 ± 11.85	-1.910	0.850
PaCO_2_ (mmHg)	66.28 ± 21.86	86.14 ± 28.31	-2.217	0.034
Etiology				
* M. pneumoniae*	6 (42.9%)	7 (33.3%)	0.326	0.413
Adenovirus	2 (14.3%)	5 (23.8%)	0.476	0.406
Type A/B influenza virus	3 (21.4%)	6 (28.6%)	0.224	0.474
* S. pneumoniae*	1 (7.1%)	1 (4.8%)	0.088	0.647
* S. aureus*	0 (0)	2 (9.5%)	1.414	0.353
Cytomegalovirus	2 (14.3%)	2 (9.5%)	0.188	0.530
Unknown etiology	6 (42.9%)	7 (33.3%)	0.326	0.413

Note: WBC, CRP, procalcitonin, blood lactate, PaO_2_, and PaCO_2_ were taken within 24 h before starting mechanical ventilation

### Comparison of sleep conditions between normal children and patients in Phases 1 and 2

**Fig. 2:**
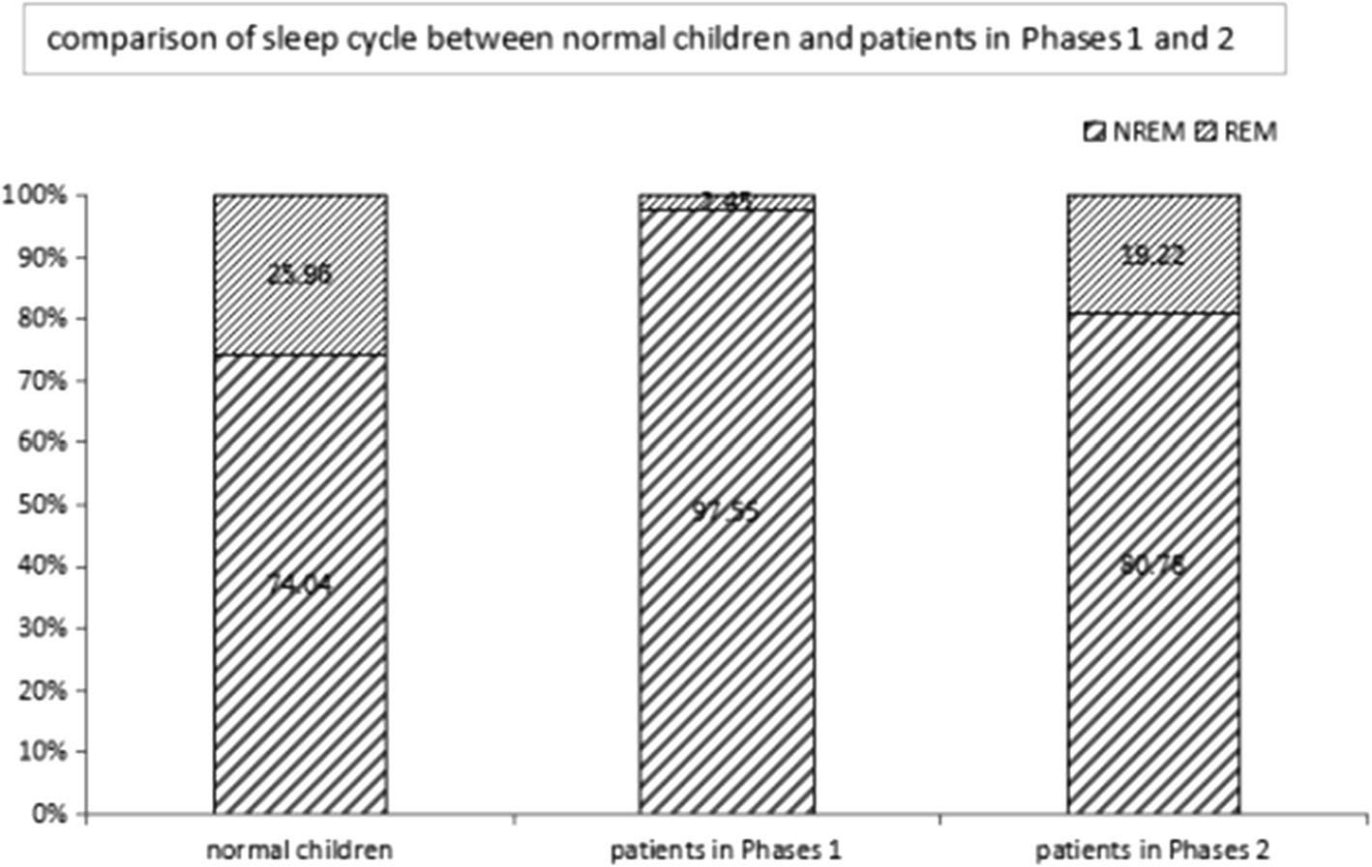
Comparison of sleep cycle between normal children and patients in Phases 1 and 2, a significant REM sleep loss occurred in phases 1 patients and the sleep cycle recovered in phases 2 patients

Among the healthy control children, there was no NREM-1 and/or REM stage loss, with REM accounting for 25.96% of the entire sleep cycle; moreover, the average sleep duration was 10.3 ± 1.2 h. The total sleep duration in a 24-h time frame was longer in Phase 1 patients (17.15 ± 2.15 h) than in the control group and Phase 2 patients (*p* < 0.05), and the sleep structure has changed significantly. Among Phase 1 patients, there were 31 (88.5%) cases of loss of NREM-1 and/or REM stage sleep, which significantly differed from the proportion among Phase 2 patients (*p* < 0.05). There was no significant difference in the sleep duration within a 24-h time frame between Phase 2 patients (9.0 ± 0.69 h) and the control group (*p* > 0.05); however, the latter was dominated by day sleep (22/32, 68.75%). The sleep cycle was normal in 27 (84.4%) cases, without loss of the NREM-1 or REM stages (Fig. 2).

### Between-group comparison of sleep status in Phase 1 based on sedation depth

**Fig. 3:**
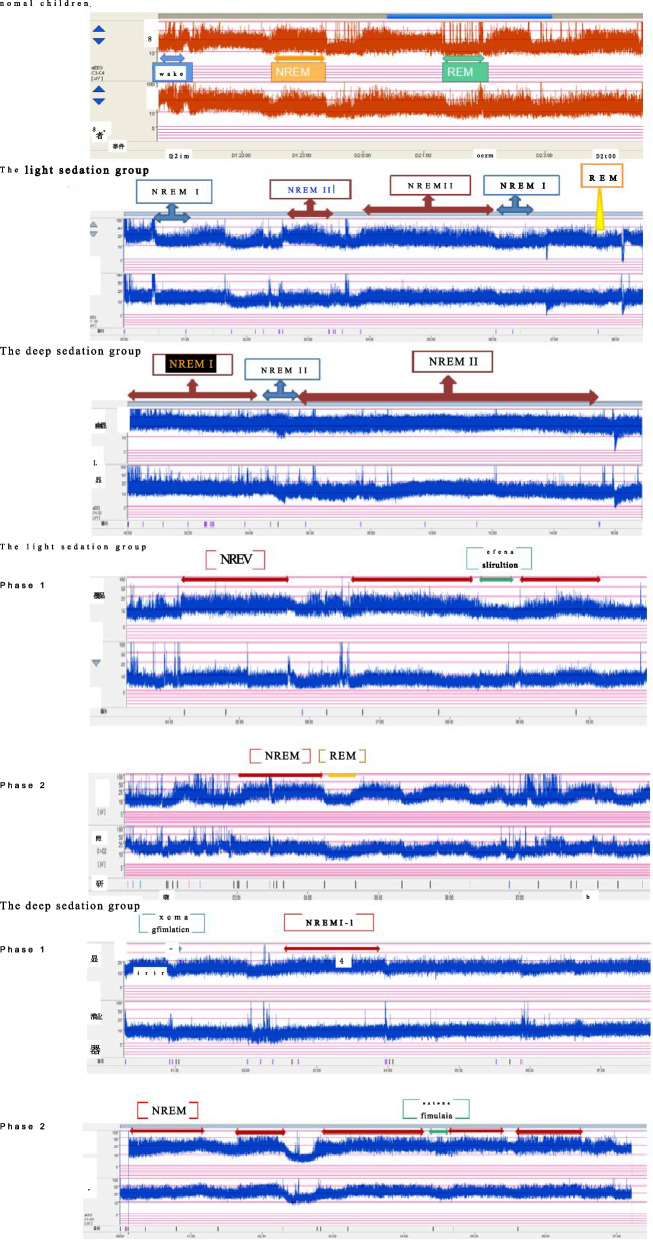
A typical case of shallow sedation group was 4-year-old boy diagnosed acute severe bronchopneumonia for 5 days, type II respiratory failure, mechanical ventilation 66 h, ramsay score 3 points, his sleep was primarily in the NREM-2 and NREM-3 stage; The typical case in the deep sedation group was 4-year-old boy, diagnosed with acute severe bronchial pneumonia for 3 days, type II respiratory failure, mechanical ventilation 74 h, ramsay score 5 points, his sleep was primarily in the NREM-3 stage. There was loss of the NREM-1 and REM stages. In Phase 1, patients in both groups experienced transient EEG changes after nursing stimulation and then entered the NREM sleep stage. In Phase 2, patients in the light sedation group basically returned to normal sleep cycles, while patients in the deep sedation group still had decreased REM sleep

Compared with the light sedation group, the deep sedation group showed a significantly higher total sleep duration, sleep efficiency, and incidence of sleep architecture altered (57.1% vs. 100%) (all *p* < 0.05). The sleep of both groups was loss of the NREM-1 and REM stages. Compared with the light sedation group, the deep sedation group had a significantly higher incidence of NREM-1 and REM stage loss and a higher proportion of NREM-3 stage (*p* < 0.05) (Fig. 3). The number of nursing care events (including lung suction, puncture and blood collection, infusion cleannessand other daily care events) in the deep sedation group was significantly higher than that in the light sedation group, with fewer awakenings (*p* < 0.05). Pearson’s correlation analysis revealed a correlation of the number of awakenings and nursing care events between both groups (*r* = 30.393, *p* < 0.05) (Table 2).

**Table 2:** Sleep status in children with different sedation depths in Phase 1

Group	Light sedation group (*n* = 14)	Deep sedation group (*n* = 21)	Test statistic (T/χ^2^)	*P*-value
Total sleep duration (h)	16.19 ± 2.30	17.52 ± 1.58	-2.292	0.028
SE (%)	67.46 ± 9.60	74.17 ± 7.62	-2.292	0.028
Abnormal sleep cycle (n) (%)	8 (57.1%)	21 (100%)	10.862	0.001
NREM-1 loss (n) (%)	10 (71.4%)	21 (100%)	6.774	0.009
N2% (%)	32.57 ± 18.68	16 ± 12.16	2.922	0.008
N3% (%)	61.07 ± 20.15	83.10 ± 12.55	-3.644	0.020
REM loss (n) (%)	14 (50%)	15 (71.4%)	1.652	0.199
Awakening	20.21 ± 4.93	14.10 ± 2.79	4.213	0.027
Nursing care events	18.9 ± 2.67	26.9 ± 4.98	-6.125	0.032

### Between-group comparison of sleep status in Phase 2 based on sedation depth

Among the 35 children, 32 children completed sleep monitoring after weaning from mechanical ventilation and sedative discontinuation. Among the remaining three children, two died during mechanical ventilation and one withdrew from the study after weaning. In Phase 2, there was no significant between-group difference in the total sleep duration (*p* > 0.05). There was a decrease in night sleep in both groups. The decrease in night sleep duration was significantly greater in the deep sedation group than in the light sedation group (*p* < 0.05). The sleep architecture in both groups indicated decreased NREM-3 and REM sleep. The decrease in REM sleep was significantly greater in the deep sedation group than in the light sedation group (*p* < 0.05). There was relatively no between-group difference in the number of night nursing care events (8, 5–11). There were significantly fewer awakenings in the deep sedation group than in the light sedation group (*p* < 0.05). Pearson correlation analysis revealed no correlation of the number of awakenings and nursing care events between both groups (*r* = 0.55, *p* > 0.05) (Tables 3 and 4).

**Table 3:** Sleep status in the two groups of children in Phase 2

Group	Light sedation group (*n* = 14)	Deep sedation group (*n* = 21)	Test statistic (T/χ^2^)	*P*-value
Total sleep duration(h)	9.19 ± 0.84	9.88 ± 1.74	1.274	0.061
SE (%)	38.28 ± 3.52	36.11 ± 3.13	1.839	0.796
Abnormal sleep cycle (n) (%)	2 (14.3%)	6 (33.3%)	1.524	0.412
N1% (%)	4.76 ± 1.96	9.89 ± 1.74	-5.191	0.788
N2% (%)	55.58 ± 11.04	62.06 ± 4.96	-2.040	0.001
N3% (%)	20.16 ± 4.85	17.06 ± 4.83	0.667	0.082
REM% (%)	20.13 ± 7.25	11.39 ± 2.12	4.367	0.001
Sleep duration at night(h)	4.86 ± 1.95	3.78 ± 1.18	1.820	0.039
Awakening	8.92 ± 0.99	9.89 ± 1.77	-1.807	< 0.05
Nursing care events	8.43 ± 3.081	9.11 ± 4.19	-5.11	> 0.05

### Between-group comparison of sleep recovery based on sedation depth

All 21 children in the deep sedation group had sleep architecture altered in Phase 1, with 6 (33.3%) children still having sleep architecture altered in Phase 2. On the other hand, 8 (57.1%) children in the light sedation group had sleep architecture altered in Phase 1, with 2 (14.28%) children still having sleep architecture altered in Phase 2 (Fig. 3). Compared with the deep sedation group, the light sedation group exhibited better sleep cycle recovery in the first 24 h after weaning from mechanical ventilation and discontinuation of sedatives and analgesics (Table 4).

**Table 4:** Between-group comparison of sleep cycle recovery

	Patients	Patients with abnormal sleep cycle in Phase 1	Patients with abnormal sleep cycle in Phase 2	Proportion with improvement
Deep sedation group	18	18 (100%)	6 (33.3%)	66.67%
Light sedation group	14	8 (57.1%)	2 (14.28%)	75.00%

## Discussion

This study observed the effects of different sedation depths on sleep among children with pneumonia-related respiratory failure without organic CNS injury. This is indicative of individual differences in the severity of genetic disease, patient-ventilator synchrony during mechanical ventilation, and required sedation for organ protection. Moreover, it suggests that appropriately light sedation is conducive for normal sleep recovery during mechanical ventilation and after weaning. EEG monitoring and sleep assessment is helpful for physicians to better implement different sedation regimens for different children. There are currently no monitoring methods appropriate for children with common sleep disorders in the PICU [17]. Long-term Video EEG is crucial and widely used for brain function evaluation in the PICU. Moreover, the use of video EEG for sleep monitoring and evaluation in children under sedation has increased knowledge regarding the actual sleep conditions of children on mechanical ventilation and under sedation. Comparison of sleep recovery after discontinuing sedatives and weaning from mechanical ventilation has also allowed more accurate sedation implementation. There is a need for sleep monitoring equipment suitable for wide use in the PICU and sleep improvement measures for critically ill children, which could improve their intra-treatment comfort and prognosis.

To show the sedation effects on the sleep cycle of pediatric patients with severe pneumonia underwent mechanical ventilation, we excluded children with CNS infection before starting mechanical ventilation or an altered state of consciousness upon admission. Studies on adult patients on mechanical ventilation showed they lack normal sleep and have frequent sleep interruptions [18]. We found that children on mechanical ventilation had prolonged sleep duration and fewer awakenings while under sedation, which is consistent with the findings by Jean et al*.* on adults [19]. Although sedative use increases sleep duration in children, it alters sleep architecture, and therefore reduces sleep efficiency. In our study, children on mechanical ventilation under sedation had slower EEG activity during sleep, diffuse δ wave activity in both hemispheres, and loss of the NREM-1 and REM stages. The deep sedation group showed more significant changes in the sleep architecture, a higher proportion of REM stage loss, and a greater decrease in the NREM-2 stage than the light sedation group. This is consistent with a previous report that benzodiazepines could decrease REM and increase NREM-2 sleep, respectively, in children on mechanical ventilation [20]. Our participants included mostly infants and young children, whose sleep regulatory mechanisms remain to be fully developed and who may experience more pronounced sedative effects on sleep. In these children, deep sedation rapidly compressed the NREM-1 and NREM-2 stages into a deep sleep stage; moreover, post-arousal sleep interruption causes decreased REM sleep. On the other hand, we included patients with severe acute pneumonia and respiratory failure, which were mostly related to hypoxemia and hypercapnia at the start of mechanical ventilation. Both hypoxemia and hypercapnia increased exhalation effort and increased arousal and therefore reduced REM sleep. Although there was no between-group difference in the Pediatric Logistic Organ Dysfunction-2 score and overall disease severity, the deep sedation group showed a significantly longer duration of mechanical ventilation and sedation than the light sedation group. This indicates no clear causal relationship between sedation depth and the duration of mechanical ventilation. However, compared with the light sedation group, the deep sedation group showed a significantly higher CO_2_ level from arterial blood gas analysis before mechanical ventilation, which is indicative of more severe pulmonary dysfunction. This could have attributed to the increased need for sedation in the deep sedation group. In this cohort study, the sedation depth was determined based on the clinical needs of the patients. This could explain why the deep sedation group had poor tolerance to mechanical ventilation, more severe lung disease, and a relatively long duration of mechanical ventilation and sedation. However, this does not exclude the actual sedation depth and duration as factors for delaying respiratory function improvement. Future studies should address these questions.

In addition to frequent awakenings and abnormal sleep architecture, abnormal circadian rhythms are among the sleep disorder manifestations in PICU patients. EEG monitoring of children on mechanical ventilation in the PICU has revealed lower daytime than nighttime slow-wave activity and reduced nighttime sleep efficiency [21]. In our study, night sleep duration in children on mechanical ventilation under sedation was significantly reduced. The total sleep duration normalized within 1 day after weaning from mechanical ventilation and sedative discontinuation; however, the night sleep duration accounted for only 40–50% of the total sleep duration. Circadian rhythm disturbances in children can cause decreased ventilation and dysfunction in the immune and endocrine systems [22]. Critically ill adult patients with severe REM sleep deprivation and interrupted circadian sleep cycles are more likely to develop delirium. Abnormal sleep cycles and structures in critically ill patients prolong the duration of mechanical ventilation and increase sedative-related short- and long-term psychiatric abnormalities [22].

Nursing care procedures are crucial for treating critically ill patients in the PICU; however, frequent nursing care procedures increase pain and stress and affect the sleep architecture. Studies have shown that adult ICU inpatients suffer from sleep interruptions while under nursing care, with increased NREM-1 stage sleep and decreased NREM-3 and REM stage sleep. In our study, the deep sedation group had more nursing care events and fewer awakenings during mechanical ventilation. In both groups, sedation depth was inversely associated with the effects of nursing care stimulation on sleep. Deep sedation in the acute phase can reduce stress response and sleep interruptions caused by stimulation. Additionally, it inhibits airway protective responses, reduces expectoration, increases airway secretions, and increases the requirement for sputum suctioning and other nursing care procedures [16, 23–25]. This explains the higher number of nursing care events in the deep sedation group than in the light sedation group. Although light sedation facilitates brain function evaluation in patients, it is more likely to cause patient-ventilator dyssynchrony and accidental extubation during mechanical ventilation. For critically ill patients requiring frequent nursing care procedures, a corresponding level of deep sedation is recommended. Although deep sedation has a greater effect on sleep architecture, it can reduce the stress response and decrease sleep interruptions caused by nursing care stimulation.

The correlation between sleep and prognosis in critically ill patients remains unclear. Sleep disorders in critically ill patients may lead to an increased delirium incidence, prolonged mechanical ventilation, and immune dysfunction. Friese et al*.* suggested that sleep deprivation can increase mortality after severe infections in mice [26]. In critically ill children whose neurocognition is still developing, the adverse effects of sleep disorders are dependent on the maturity of the sleep arousal regulation system and the overall neurological development stage. Moreover, there is more persistent damage to myelination, synapse formation, or emotional control regulation at younger ages. Sleep spindles and K-complexes appear in NREM-2 sleep, which is a crucial stage for memory integration and maintenance. Boyko et al*.*^*7*^ confirmed that loss of sleep spindles in adults can increase the mortality risk. Most children in this study resumed their sleep cycles after weaning from mechanical ventilation and sedative discontinuation; however, their circadian rhythms remained abnormal. This is related to disease and environmental factors in the ICU; moreover, sleep deprivation and abnormal sleep cycles that occur in the ICU can persist for a few post-discharge months and affect prognosis. Whether children on mechanical ventilation in the PICU develop sleep disorders after discharge remains unclear.

Compared with sleep disorders in adults, those in children are often overlooked. There has been a recent increase in attention on these sleep disorders. including the use of melatonin for sleep disorders in children with neuromuscular diseases [27] and respiratory sleep disorders [28] and sleep changes during muscle relaxant treatment in the PICU. It is difficult to identify sleep changes in children with pneumonia and comorbid organic nervous system damage. Sleep changes in children without neurological disease, especially after sedation during mechanical ventilation, remain unclear; therefore, we conducted this study. This study has a number of limitations. Many ICU patients are on mechanical ventilation; however, given the relatively young age of our patients, the leads fell off during the trial recording period for some patients, or the monitoring was interrupted for other reasons, which resulted in mid-study withdrawal and a relatively small sample size. Additionally, sleep may be affected by procedures conducted by medical and nursing personnel in the PICU, the light and sounds in the ward, the ventilator mode, and other factors. Follow-up studies should investigate the effects of different ventilator modes on patients and effects of mild hypothermia treatments on sleep, as well as expand the scope of application and monitoring.

## Conclusions

In this study, children with severe acute bronchopneumonia who received invasive mechanical ventilation under sedation showed EEG abnormalities. These were characterized by decreased brain electrical activity, sleep periodicity, and reduced/absent physiological sleep waves. Deep sedation during mechanical ventilation can cause longer total sleep duration, shorter awakenings, and increased proportion of deep sleep; however, it may worsen abnormal sleep architecture. After weaning from mechanical ventilation and sedative discontinuation, lightly sedated children exhibited better sleep cycle recovery. Future studies should elucidate the long-term physical and psychiatric effects.

## Supplementary Information

**Additional file 1:Supplemental Table 1.** Ramsay sedation scale. **Supplemental Table 2.** American Academy of Sleep Medicine (AASM) sleep score.

## Data Availability

Supporting data from this study can be obtained by emailing the corresponding author.

## Abbreviations

ICU: Intensive Care Unit
PICU: Pediatric Intensive Care Unit
EEG: Electroencephalography
NREM: Non-rapid eye movement sleep
REM: Rapid eye movement sleep
ARDS: Acute respiratory distress syndrome
CNS: Central nervous system
BIPAP: Biphasic positive airway pressure
AASM: American Academy of Sleep Medicine
SD: Standard deviation
