# Supplementary material for: Sleep cycle in children with severe acute bronchopneumonia during mechanical ventilation at different depths of sedation
Source: BMC Pediatr. 2022 Oct 12;22:589. doi: 10.1186/s12887-022-03658-8 (PMC9553625; doi:10.1186/s12887-022-03658-8)
Supplement: Supplementary file 1 — Additional file 1:Supplemental Table 1. Ramsay sedation scale. Supplemental Table 2. American Academy of Sleep Medicine (AASM) sleep score. [file 12887_2022_3658_MOESM1_ESM.doc]

Supplemental Table 1. Ramsay sedation scale.

| Score | Clinical presentation |
| --- | --- |
| 1 | Anxiety, agitation, restlessness |
| 2 | Cooperative, tranquil, well oriented, and tolerating mechanical ventilation well |
| 3 | Only responds to commands |
| 4 | Exhibits brisk response to light glabellar tap or loud auditory stimuli |
| 5 | Exhibits sluggish response to light glabellar tap or loud auditory stimuli, without responding to painful stimuli |
| 6 | Exhibits no response to light glabellar tap or loud auditory stimuli |

Supplemental Table 2. American Academy of Sleep Medicine (AASM) sleep score.

| AASM criteria for scoring sleep | |
| --- | --- |
| NREM-1 | Predominantly theta, possible vertex sharp waves, possible slow eye movements |
| NREM-2 | Sleep spindles and/or K-complexes not associated with arousal |
| NREM-3 | Delta ≥ 20% of the epoch |
| REM | Low amplitude, mixed frequency EEG pattern, sawtooth waves, rapid eye movements, low sub-mental tone |
| Wake | Alpha, eye blinks, reading eye movements, rapid eye movements, normal or high sub-mental tone |

Note: NREM, non-rapid eye movement sleep; REM, rapid eye movement sleep.
